# New-designed 3D printed surgical guide promotes the accuracy of endodontic microsurgery: a study of 14 upper anterior teeth

**DOI:** 10.1038/s41598-023-42767-x

**Published:** 2023-09-19

**Authors:** Dan Zhao, Weige Xie, Tianguo Li, Anqi Wang, Li Wu, Wen Kang, Lu Wang, Shiliang Guo, Xuna Tang, Sijing Xie

**Affiliations:** 1 Department of Endodontics, Nanjing Stomatological Hospital, Affiliated Hospital of Medical School, Nanjing University, Nanjing, 210008 Jiangsu People’s Republic of China; 2 Nanjing Tongren Hospital, Nanjing, 210008 Jiangsu People’s Republic of China; 3 Nantong Stomatological Hospital, The Affiliated Nantong Stomatological Hospital of Nantong University, Nantong, 226000 Jiangsu People’s Republic of China

**Keywords:** Dental diseases, Endodontics

## Abstract

We aimed to design a novel three-dimensional (3D) printed surgical guide and evaluate its accuracy in assisting endodontic microsurgeries. A new 3D printed surgical guide was designed by computer-aided design and computer-aided manufacturing (CAD/CAM) technology and applied to 7 patients who underwent endodontic microsurgeries of upper anterior teeth from 2020.01 to 2020.12 as the experimental group. 7 patients who suffered from endodontic microsurgeries operated by the same surgeon without using the surgical guide from 2019.01 to 2019.12 were selected as the control group. Cone beam computed tomography (CBCT) was performed more than 12 months after operation, and the accuracy of apical resection was compared between the two groups. The accuracy of the microsurgery focused on the length and angle of the root apical resection. In the study, CBCT data and oral digital scanning data were used to reconstruct 3D models of periapical lesions with soft and hard tissue information, based on which we designed the new 3D printed surgical guides. The guides were successfully applied to the apectomy in endodontic microsurgeries. The deviation of the apical resection length of the experimental group (0.467 ± 0.146 mm) was better than that of the control group (1.743 ± 0.122 mm) (P < 0.0001), and the deviation of the apical resection angle of the experimental group (9.711 ± 3.593°) was significantly less than that of the control group (22.400 ± 3.362°) (P < 0.0001). The 3D-printed surgical guide could effectively guide endodontic microsurgery and improve its accuracy by fixing both the position and the angle of apectomy. The new type of surgical guide could accurately localize the root apex and guide the apical resection.

## Introduction

Chronic periapical cysts or cases with substantial destruction of the periapical area usually require endodontic microsurgery to remove periapical inflammation and promote tissue healing^1^. Compared with traditional therapy, the success rate of modern endodontic microsurgery reaches up to 89%^2^. To minimize the possibility of microleakage and preserve the remaining dental tissue to the greatest extent, apical resection is the key step in endodontic microsurgery^3–5^. However, it is difficult for the surgeon to achieve precise apical resection due to the limited surgery field clinically.

With the development of modern digital technologies such as 3D imaging, CBCT, and CAD/CAM, there have been some achievements in the clinical application of 3D printed surgical guides in apical surgery, realizing the precise positioning of the root apical lesion area, the reduction of surgical trauma and the protection of adjacent tissue structure to a certain extent^6–8^. At present, research on digitally guided endodontic microsurgery mainly focuses on individual case reports^9–11^. In the current studies, there are 3 types of apical surgery assisting guides: localized bone-removing guide, fixed-depth bone-removing guide, and localized fixed-depth bone-removing guide^12–18^. These kinds of guides provide a significant improvement in bone removal^19^, but none of them could achieve precise resection of the root apex in length and angle, which is more valuable in apical surgery. Previous studies mainly focused on clinical qualitative indicators, such as the long-term outcomes of apical surgery and the survival and healing status of the target teeth^20^. A previous in vitro study by the same research group evaluated the operative accuracy of digitally guided endodontic microsurgery and confirmed that the length and angular deviation of the resection was significantly reduced by surgical guide^21^. However, no clinical research has confirmed the accuracy of digitally guided endodontic microsurgery.

Our study reconstructed the periapical lesion models from CBCT data and oral digital scanning data and designed the novel 3D printed metal surgical guide based on apical surgery simulation, localized digital guide manufacture, and image fitting comparison. The new type of surgical guide could accurately localize the root apex and guide the apical resection.

## Materials and methods

### Design and manufacture of 3D printed surgical guide

**Figure 1:**
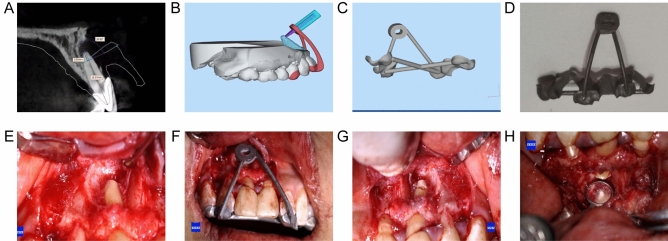
The design process and clinical application in endodontic microsurgery. (**A**) CBCT data of and the virtual bur data were merged in the designning software. (**B**) Establish the long axis of the target tooth and build the virtual bur-cutting trajectory. (**C**) Virtual model of 3D printed surgical guide. (**D**) Material object of 3D printed surgical guide. (**E**) The full-thickness mucoperiosteal flap was raised and the root apex and cyst wall were exposed. (**F**) The guide was aligned in position. (**G**) Root apex was extracted under the guidance of the 3D printed guide. (**H**) iRoot BP was backfilled into the cavity after bleeding control.

After plaster infusion with alginate and optical scanning, we obtained the study model of the patient. The model was converted into digital data and exported in STL format. The patients’ preoperative CBCT data were taken and the DICOM files obtained before the operation were imported into Mimics Research 20.0 for image segmentation and 3D reconstruction. In Mimics Research 20.0, threshold segmentation was used to establish a 3D reconstruction model of the bone defect of periapical disease. The maxillary bone and mandibular bone were segmented by Geomagic Studio and merged with the optically scanned dentition data. The model was saved and exported in STL format. We established the long axis of the target tooth according to the simulated periapical lesion model, and the virtual bur-cutting trajectory is built. The guide was designed to precisely resect 3 mm of the apex and be perpendicular to the long axis of the tooth. In addition, a window was designed on the guide, matching the position of the periapical lesion, and guiding the access to the exact location of the apical surgery. Once the design was complete, the surgical guide was exported as an STL file and was 3D printed with nichrome following the manufacturer’s guidelines. (Fig. 1A–D).

### Case selection

The objects of the study were patients aged 18 to 45 who underwent endodontic microsurgery on the upper anterior teeth with the presence of large and intruding periapical lesions after relatively complete root canal filling. Every participant was informed of the therapeutic schemes and signed written informed consent form prior to beginning the study. The patients who were unwilling to participate the research, or the clinical data was incomplete and the patients with systemic disease or chronic periodontitis were excluded. 7 patients who underwent endodontic microsurgeries using 3D printed surgical guides from 2020.01 to 2020.12 were selected as the experimental group. And another 7 patients who suffered from endodontic microsurgeries operated by the same surgeon without a surgical guide using from 2019.01 to 2019.12 were selected as the control group. The study was approved by the Institutional Review Board and all methods were performed in accordance with the relevant guidelines and regulations.

### Endodontic microsurgery using 3D printed surgical guide

All operations were performed by the same physician under the microscope. Two weeks after root canal therapy, the endodontic microsurgery was performed under local anesthesia (4% articaine with 1:100,000 epinephrine). A full-thickness mucoperiosteal flap was raised and the buccal bones were removed with a round bur under continuous saline irrigation to access the root apex. Positioned the 3D-printed surgical guide on the targeted tooth, and checked its stability and adaptation. After removing all inflammatory tissues, completely removed the root apex of about 3 mm perpendicular to the long axis of the tooth under the guidance of the guide with a carborundum bur TF11. Backfilled the apical canal with iRoot BP after bleeding control. Finally, the full-thickness flap was repositioned and sutured. (Fig. 1E–H) Took out stitches after 7–10 days. The experimental groups underwent endodontic microsurgeries according to the aforementioned procedures: model reconstruction of periapical lesions, 3D printed surgical guides design and surgery performance under the guide. While the control groups underwent a routine endodontic microsurgical procedure to complete the operations. All the operations were performed by the same physician.

### Post-operation follow-up

**Figure 2:**
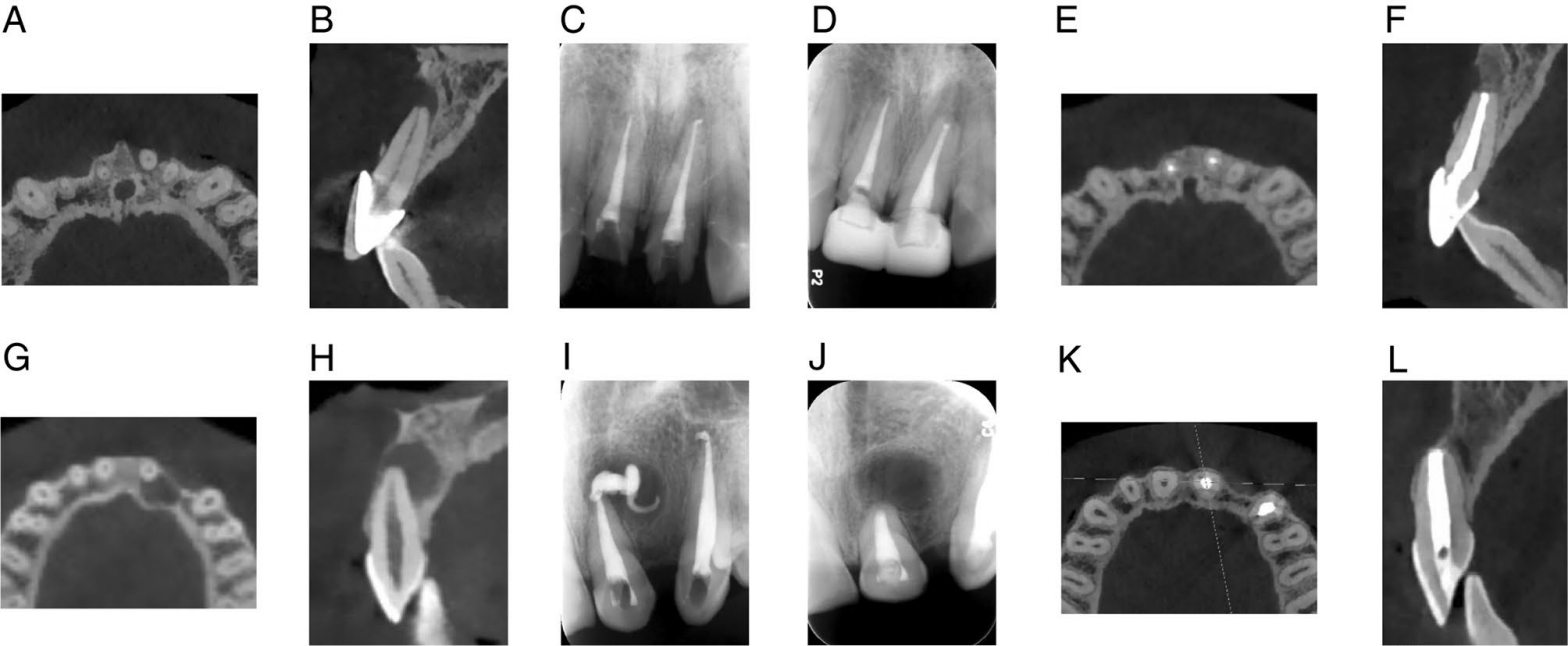
Two cases of endodontic microsurgery with 3D printed surgical guide. Case 1: Preoperative radiographic imaging (**A**,**B**), periapical radiograph after root canal treatment (**C**), periapical radiograph right after microsurgery (**D**) and radiographic imaging recalled after 12 months (**E**,**F**). Case 2: Preoperative radiographic imaging (**G**,**H**), periapical radiograph after root canal treatment (**I**), periapical radiograph right after microsurgery (**J**) and radiographic imaging recalled after 12 months (**K**,**L**).

Patients were recalled after 7 days for clinical examination and after 12 months for radiographic examination (Fig. 2). Symptoms of palpation or percussion discomfort, sinus tracts, and tooth mobility were recorded. Periapical radiographs of at least 12 months were assessed and independently assessed by two examiners according to Molven's criteria. Each case was assessed as one of the following: complete healing, incomplete healing, indeterminate healing, and unsatisfactory healing. Treatment outcomes were based on clinical and radiographic assessments. A successful outcome was accounted for in the absence of clinical signs or symptoms of apical periodontitis accompany by radiographic evidence of complete healing or incomplete healing. Conversely, the presence of clinical signs or symptoms of apical periodontitis or radiographic evidence of indeterminate healing and unsatisfactory healing would be classified as a failure outcome.

### Accuracy assessment

Accuracy assessment of the data measurement index included both apex resection length deviation (RL) and apex resection angle deviation (RA).

#### Accuracy assessment of apical resection length (RL)

**Figure 3:**
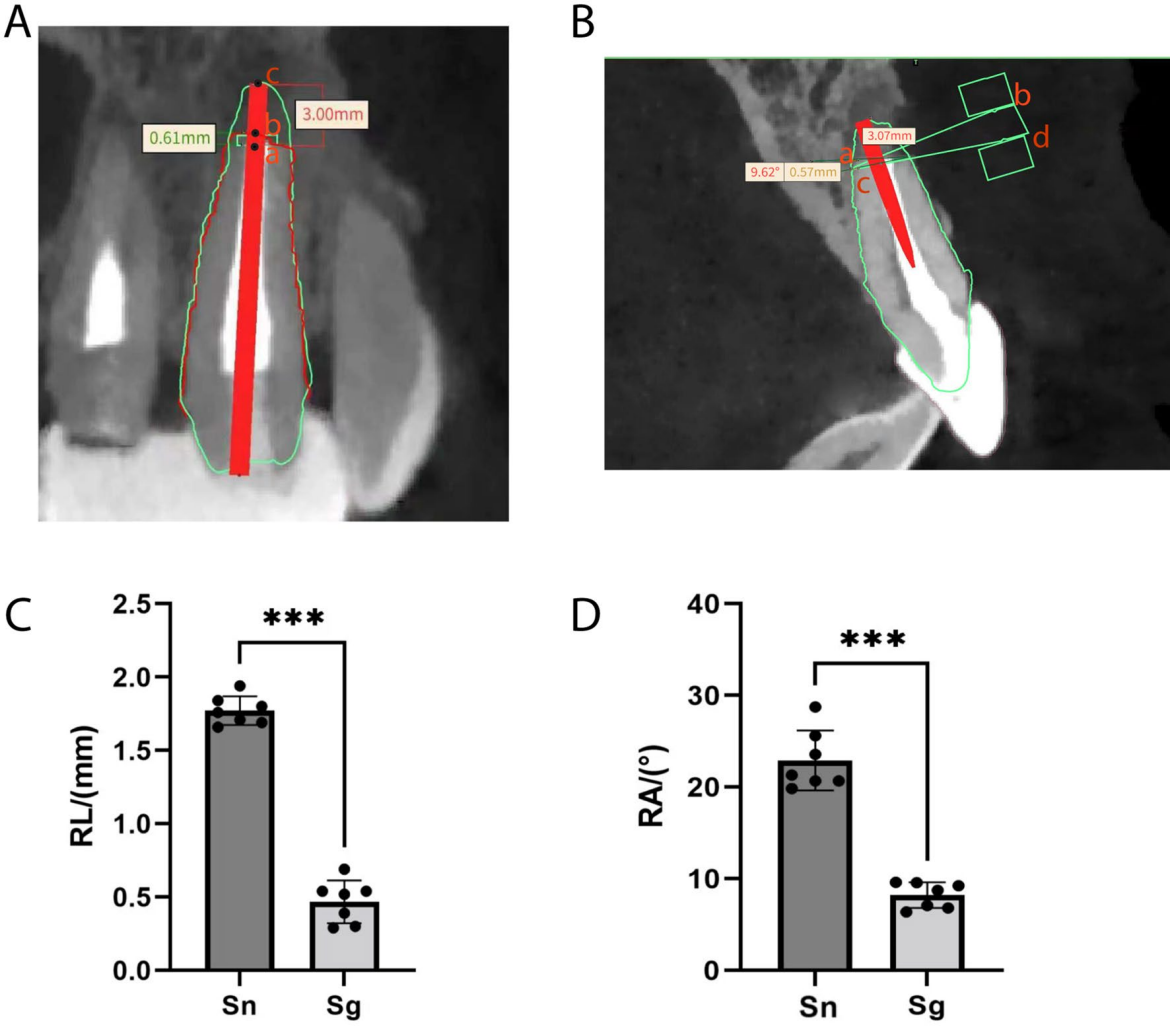
Accuracy assessment of apical resection length and apical resection angle. (**A**) Apex resection length deviation (RL) = preoperative root length (ac)—postoperative root length (ab)—preset apical resection length (3 mm). (**B**) The Angle (RA) formed by line ab and line cd is the deviation angle between the experimental apical section and the standard apical section. (**C**) The length deviation of apical resection was significantly reduced when the guide was used (P < 0.001). (**D**) The accuracy of the apical resection angle of the experimental group was better than that of the control group. (P < 0.001). (Sn: control group, Sg: experimental group).

Based on the post-operative CBCT, the data was derived in DICOM format and 3D reconstruction was performed using Mimics Research 20.0. The consistent adjacent teeth were used as a reference to overlapping the post-operative data with the preoperative data. Located the long axis of the tooth and determined the central sagittal plane of the tooth. The initial length from the incisal end to the apex of the target tooth was imported into the software to fit the post-operative data. Apex resection length deviation (RL) = preoperative root length (ac)—postoperative root length (ab)—preset apical resection length (3 mm). (Fig. 3A).

#### Accuracy assessment of apical resection angle (RA)

The ideal situation is that the apical resection plane is perpendicular to the tooth’s long axis. Line ab was the junction of the ideal section and the central sagittal plane. In other words, line ab indicated the standard apical resection plane. Similarly, the post-operative data were input to the Mimicsmedic 20.0 and adjusted to the same position. The junction of the surgical section and the central sagittal plane was called line cd. Line cd represents the actual apical resection plane. The Angle (RA) formed by line ab and line cd is the deviation angle between the experimental apical section and the standard apical section. (Fig. 3B) The standard value of RA is 0°. The larger value, the larger deviation.

### Statistical analysis

The statistic analysis was performed in SPSS 22.0 (IBM, USA), and normally distributed measures were described as x ± s. Normality tests were performed using the method of Shapiro–Wilk. The apical resection length and angle were calculated from preoperative and postoperative CBCT data. After excluding the confounding effect of the guide itself, the variables were subjected to a one-sample t-test (α = 0.05), and descriptive statistics (mean and standard deviation) was also calculated by the group.

### Ethics approval

The study was approved by the Institutional Review Board of Nanjing Stomatological Hospital (YW-2000NL-004, Nanjing, China).

### Informed consent

The written informed consent was obtained from each patient.

## Results

### The new 3D-printed surgical guide was designed and applied to endodontic microsurgeries

In the study, we used multiple digital technologies and CAD/CAM to set up a digital design method for an apical surgery guide and a new kind of metal surgical guide was successfully produced. Preliminary verification of the design route and fabrication method in 7 cases of endodontic microsurgeries of upper anterior teeth showed its feasibility. The 3D-printed surgical guide was convenient to place, with good retention and stability. It could assist the surgeon to complete the apical resection with the removal of root canal infection and fairly good effect of root apex preparation and backfilling, which could meet clinical needs.

### Post-operation follow-ups showed a high success rate of endodontic microsurgeries

The average age of the patients was 31.1 years old. The number of patients above 30 years old and those under 30 years old was almost the same, and the number of men and women was equal. The maxillary central incisors were the most (64.29%), followed by the maxillary lateral incisors (35.71%). Patients were recalled 7 days after microsurgery for clinical examination. There was only 1 patient without guide using showed pain at the 7th day. None of the two groups showed symptoms of palpation or percussion discomfort, sinus tracts, or tooth mobility during the follow-up period. The mean follow-up period of the 14 patients for radiographic examination was 18.5 months. Postoperative radiographic examination showed complete healing in 11 cases (78.57%) and incomplete healing in 3 cases (21.43%), the total success rate of the endodontic microsurgeries was 100%. (Table 1).

**Table 1:** Post-operation follow-ups in clinical (7d) and radiographic (12 m) examination.

		Control group (Sn) (n = 7)	Experimental group (Sg) (n = 7)	Proportion (100%)
Gender	Male	4	3	50%
	Female	3	4	50%
Tooth positon	Maxillary central incisor	6	5	78.57%
	Maxillary lateral incisor	1	2	21.43%
Pain	( +)	1	0	7.14%
	( −)	6	7	92.86%
Percussion	Present	0	0	0%
	Absent	7	7	100%
Mobility	Present	0	0	0%
	Absent	7	7	100%
Palpation	Present	0	0	0%
	Absent	7	7	100%
Radiographic examination	Complete healing	5	6	78.57%
	Incomplete healing	2	1	21.43%
	Uncertain healing	0	0	0%
	Unsatisfactory healing	0	0	0%

### The length deviation of apical resection of the experimental group was significantly reduced compared to the control group

Shapiro–Wilk test confirmed that the deviation of the apical resection length of the two groups was normally distributed. The one-sample t-test compared the deviation of apical resection length in the guide group (0.467 ± 0.146 mm) with that in the control group (1.743 ± 0.122 mm) showed that the length deviation of apical resection was significantly reduced when the guide was used (P < 0.001) (Table 2 and Fig. 3C).

**Table 2:** The length and angle of apical resection in the two groups.

	n	Apex resection length deviation (RL) (mm)	Apex resection angle deviation (RA) (°)
Control group (Sn)	7	1.743 ± 0.122	22.400 ± 3.362
Experimental group (Sg)	7	0.467 ± 0.146	9.711 ± 3.593
*P* value	–	< 0.001	< 0.001

### The accuracy of the apical resection angle of the experimental group was better than that of the control group

Shapiro–Wilk test showed the deviation of the apical resection angle of the two groups in a normal distribution. One-sample T-test was used to compare the deviation of apical resection angle between the experimental group (9.711 ± 3.593°) and the control group (22.400 ± 3.362°), and the discrepancy was statistically significant (P < 0.001) (Table 2 and Fig. 3D). The results showed that the accuracy of the apical resection angle of the experimental group was better than that of the control group.

## Discussion

In the process of endodontic microsurgery, accurate control of the length and angle of apical resection is very important for minimally invasive and precise surgery^22^. It is also an important factor affecting the success rate and survival rate of the target teeth^23^. Precise manipulation can reduce the risk of injury to important anatomical structures such as the inferior alveolar nerve and the maxillary sinus^24^. Modern apical surgery requires that the length of apical resection is generally 3 mm, the resection plane is perpendicular to the long axis of the tooth, and the inclination doesn’t exceed 10°^25^. Under such standard, the remaining tooth tissue could be preserved to the greatest extent with sufficient apical resection, and limited exposure of dentinal tubules, all of which can reduce the possibility of microleakage.

In our study, a series of measures were taken to ensure the surgical accuracy of endodontic microsurgery: the emergence of the hard tissue data from CBCT and the soft tissue data from digital scanning, and the reconstruction of a 3D model with both hard and soft tissue information. According to the long axis of the target tooth, a virtual cutting trajectory and the shape of the guide were established, so that the resection trajectory of the guide could reach the preset apical resection length and the resection plate could be perpendicular to the long axis of the tooth. When the DICOM file and the optical scanning file are combined to design a surgical guide, all parameters of the apical resection are determined, and once the guide is positioned correctly, the clinical deviation from the ideal designed resection would be minimal. Compared with the routine method of rough judgment and guidance based on CBCT images, the application of the surgical guide for apical resection makes apical resection more intuitive and controllable.

In the previous literatures, most surgical guides were made of polymer materials such as resin or polylactide. Many researchers had discussed the drawback of polymer guides. Polymer guides need to be designed relatively thick to ensure the strength. The hardness of polymer materials is less than that of high-speed bur, which would lead to guide abrasion and loss of surgical accuracy^26^. Numbers of polymer guides need incorporated sleeves to help guide the bur, which raise the related risks such as dislocation between guide and sleeve^27^. Some researchers have also proposed that there would be possible contamination with plastic particles during drilling with polymer guides^17^. Considering the above factors, we selected metal as the material.

The study by Fan et al. found that the deviation of apical resection guided by grid position was 0.66 ± 0.54 mm, and the deviation of apical resection without a guide was 1.92 ± 1.05 mm^28^. The randomized controlled study by Ackerman et al. reported that the deviation of apical resection with the assistance of a 3D printing guide was 1.473 ± 0.751 mm, and the deviation of apical resection in the unguided group was 2.638 ± 1.387 mm^21^. The previous in vitro research of our group confirmed that the 3D-printed apical surgical guide played a big role in apical resection depth and angle, which improved the accuracy of surgery and reduced the technical sensitivity^29^. Compared with the group without the guide, the apical resection length of the guide group was close to the current standard of 3 mm (P < 0.05). All the above literature shows that the application of the digital guide can assist the operator in accurately removing the apical lesions, improve the accuracy of the surgery, minimize the damage of the soft and hard tissues around the operation area, reduce the surgery risk, minimize the intraoperative and postoperative complications and improve the success rate of the apical surgery^30,31^.

However, there has been little quantitative analysis of the feasibility and accuracy of 3D-printed surgical guides in clinical practice. Our study describes cases of endodontic microsurgeries with 3D-printed surgical guides and compares the accuracy to the operations without a guide. From the results of the analysis of the accuracy of the guide-assisted apical resection, the length of the guided apical resection is more approach 3 mm, and the angle between the apical resection section and the ideal cross-section is nearly 0°. Both the section length and angle have been significantly improved. The results suggest that guided apical localization and resection can be performed in the cases of apical surgery. The duration of root resection is also an important clinical indicator. The previous in vitro research of our group had included the impact of the guide on the duration of root resection^29^. We found that 3D printed surgical guide could shorten the operation time of junior physicians, but the operation time of senior physicians didn’t show significant difference. For the operator of this research is a senior physician, we didn’t include the indicator of root resection time. But the application of 3D printed surgical guide would be valuable to junior physicians. This study provides a more precise surgical method for apical resection, complete apical preparation and backfilling, removing infection, and preventing reinfection. In the future, it will make greater clinical significance as an application to posterior teeth.

This research mainly focuses on the accuracy of apical resection assisted by digital guides. The follow-up period is not sufficient to evaluate the clinical success and long-term prognosis. Our group is planning to carry out long-term follow-ups of endodontic microsurgeries to move forward with a single step. There was only one operator in the study, which may lead to biases in the effect of clinical experience on the accuracy of apical resection. To improve the accuracy of microscopic apical surgery by different doctors, further clinical training and research are needed. Accuracy assessment based on the target tooth position may also be a key point for preoperative preparation of endodontic microsurgeries.

## Conclusion

The newly designed 3D-printed surgical guide could effectively assist endodontic microsurgery and improve its accuracy by fixing both the position and the angle of apectomy.

## Data Availability

The datasets used and analysed during the current study are available from the corresponding author on reasonable request.
